# A phase I trial of high-dose palliative radiotherapy plus concurrent weekly Vinorelbine and Cisplatin in patients with locally advanced and metastatic NSCLC

**DOI:** 10.1038/sj.bjc.6602759

**Published:** 2005-09-06

**Authors:** M Michael, A Wirth, D L Ball, M MacManus, D Rischin, L Mileshkin, B Solomon, J McKendrick, A D Milner

**Affiliations:** 1 The Division of Haematology and Medical Oncology, Peter MacCallum Cancer Centre, Locked Bag 1, A'Beckett St, Victoria 8006, Australia; 2 The Division of Radiation Oncology, Peter MacCallum Cancer Centre, Locked Bag 1, A'Beckett St, Victoria 8006, Australia; 3 Department of Medical Oncology, Box Hill Hospital, Nelson Rd, Box Hill, Victoria 3128, Australia; 4 Centre for Biostatistics and Clinical Trials, Peter MacCallum Cancer Centre, Locked Bag 1, A'Beckett St, Victoria 8006, Australia

**Keywords:** lung cancer, high-dose palliative radiotherapy, chemoradiotherapy, palliative benefit

## Abstract

The role of concurrent chemoradiotherapy (CRT) in patients with non-small-cell lung cancer (NSCLC) unsuitable for radical therapy but who require locoregional treatment has not been defined. The aims of this phase I trial were thus to develop a novel regimen of weekly chemotherapy concurrent with high-dose palliative RT (40 Gy/20 fractions) and assess its tolerability, objective and symptomatic response rates. Eligible patients had stage I–IIIB NSCLC unsuitable for radical RT or limited stage IV disease, ECOG PS⩽1 and required locoregional therapy. Treatment was RT (40 Gy/20 fractions/5 per week) and weekly Vinorelbine plus Cisplatin escalated in six planned dose levels (DLs). At 4 weeks post-RT, patients received two cycles of Cisplatin 80 mg m^−2^ day 1+Vinorelbine 25 mg m^−2^ days 1, 8, 15. Dose-limiting toxicities (DLTs) were defined in the CRT phase. Disease-related symptoms were assessed by the Lung Cancer Symptom Scale. In all, 24 patients accrued, stage IIIB (*n*=12) and IV disease (*n*=10). The highest administered dose was at DL 4, Vinorelbine 30 mg m^−2^+Cisplatin 20 mg m^−2^ with DLTs of grade 4 neutropenia in two of three patients. No grade 3 or 4 nonhaematological toxicities were observed. The overall radiological response rate was 65% (*n*=23: complete response 4% and partial response 61%) and infield FDG-PET responses were seen in 89% (*n*=18). There was an improvement or stabilisation of symptoms and quality of life. Dose level 3, Vinorelbine 25 mg m^−2^+Cisplatin 20 mg m^−2^, is recommended for further assessment. This regimen was tolerable and produced meaningful responses for patients for whom locoregional control is required, but who are unsuitable for radical CRT.

The majority of patients with unresectable stage IIIA or IIIB non-small-cell lung cancer (NSCLC) are precluded from radical curative chemoradiotherapy (CRT) either due to the presence of extensive intrathoracic disease, poor performance status or significant comorbidities. A subgroup who have good performance status, limited metastatic involvement and require palliation of local symptoms have been treated with various regimens of high-dose palliative radiotherapy (HDPR), ranging from 30 Gy in 10 fractions to 42 Gy in 15 fractions or equivalent (MRC Lung Cancer Working Party, 1992; Macbeth *et al*, 1996; Ball *et al*, 1997; Plataniotis *et al*, 2002; Kramer *et al*, 2003; Sundstrom *et al*, 2004). High-dose palliative radiotherapy has been shown to provide an excellent palliative benefit through the reduction of local symptoms in 80–90% of patients, and also benefits in terms of global quality of life (QOL) (Schaafsma and Coy, 2000; Bezjak *et al*, 2002; Sundstrom *et al*, 2004).

A widely utilised alternative to HDPR has been the use of hypofractionated radiation schedules, which are considerably less resource and time intensive. However, a large retrospective analysis (Quddus *et al*, 2001) as well as randomised studies have confirmed that, relative to hypofractionated regimens, HDPR in patients with good performance status provides a greater benefit in terms of local symptom palliation, global QOL and survival, possibly in a dose-dependent manner (Simpson *et al*, 1985; Teo *et al*, 1988; Macbeth *et al*, 1996; Reinfuss *et al*, 1999; Gaze *et al*, 2001; Bezjak *et al*, 2002; Kramer *et al*, 2003). These observations though have not been consistent (MRC Lung Cancer Working Party, 1991; Nestle *et al*, 2000; Sundstrom *et al*, 2004).

Can the advances seen for radical CRT be translated to the HDPR setting, where the dominant competing risk for death is locoregional disease, that is, thoracic dominant disease? Randomised trials have demonstrated that the addition of platin-based chemotherapy to radiation is associated with a survival advantage when either given as induction (Sause *et al*, 1995; Dillman *et al*, 1996) or concurrent treatment (Schaake-Koning *et al*, 1992) compared to radiation alone. It is unclear whether combining concurrent chemotherapy with HDPR offers an advantage in terms of response, palliation and survival. Two studies have been reported exploring this approach, but with hypofractionated radiation regimens associated with significant toxicities (Jeremic *et al*, 1999; Slivano *et al*, 2000).

Vinorelbine is a vinca alkaloid with activity both as a single agent and in combination with platinum analogues in patients with NSCLC (Le Chevalier *et al*, 1994; Bunn and Kelly, 1998; The Elderly Lung Cancer Vinorelbine Italian Study Group, 1999). The agent is also a radiation sensitiser (Edelstein *et al*, 1996), which has led to its assessment in combination with platinum analogues and radical radiotherapy. The radiation and chemotherapy schedules have varied between studies; however, response rates have ranged from 62.5 to 80.4% (Masters *et al*, 1998; Hoffman *et al*, 2002; Vokes *et al*, 2002; Zatloukal *et al*, 2004).

The aims of this trial were thus to determine the highest administered dose of weekly Cisplatin and Vinorelbine combined with HDPR (40 Gy) for patients with good performance status who were not suitable for radical CRT, assess the efficacy of this regimen by structural and functional imaging, and explore its effect upon disease-related symptoms (DRS). The secondary objectives were to determine the progression-free survival (PFS) and overall survival (OS) of patients treated with this regimen.

## MATERIALS AND METHODS

### Patients

Patients who met the following criteria were eligible: (1) histological or cytological proven NSCLC with measurable disease; (2) symptomatic locoregional disease; (3) unsuitable for radical CRT – examples include: (i) disease extent technically unsuitable for radical therapy, where the lung volumes encompassed by radiotherapy plans for 60 Gy were considered unacceptable, due to the need to include N3 disease (supraclavicular or contralateral mediastinal or hilar nodal involvement) or an excessive superior–inferior disease extent, (ii) poor prognostic factors such as weight loss in excess of 10%, (iii) concurrent medical illness or (iv) stage IV disease with limited extra-thoracic spread, where it is judged that the dominant competing risk for death is uncontrolled locoregional disease; (4) no prior radiotherapy or chemotherapy; (5) ECOG performance status⩽1; (6) life expectancy in excess of 3 months; (7) adequate organ function – (i) hepatic: serum bilirubin⩽1.0 × ULN, AST and/or ALT⩽2.0 × ULN, ALP and GGT⩽2.5 × ULN, if bone or liver involvement, AST or ALT, ALP and GGT⩽5.0 × ULN, (ii) bone marrow: haemoglobin⩾100 g l^−1^, neutrophil count⩾1.5 × 10^9^ l^−1^, platelet count⩾100 × 10^9^ l^−1^, (iii) renal: creatinine clearance⩾55 ml min^−1^ (using radioisotope renal scan or derived from serum creatinine using the Cockcroft–Gault formula); (8) age less than 75 years; (9) written informed consent.

The following patients were ineligible: (1) significant medical conditions which were considered to compromise the planned delivery of the chemotherapy and radiotherapy or be potentially exacerbated by these modalities; (2) history of any other cancer (except nonmelanoma skin cancer or carcinoma *in situ* of the cervix) unless in complete remission and off all therapy for that cancer for at least 5 years; (3) receiving treatment with another investigational agent. Institutional ethics committee approval was obtained.

### Treatment plan

#### Chemoradiotherapy

1. Radiation therapy: All radiotherapy was administered using 6 MeV linac, to a dose of 40 Gy in 2 Gy fractions, 5 per week for 4 weeks. The treatment plan was according to normal institutional practice, namely: (1) AP-PA treatment fields, (2) planning target volume (PTV)=gross macroscopic disease (GTV)+1 cm, (3) dose specified at the midplane on the central axis; however, regions which may receive a greater dose by virtue of variation in patient contour (e.g. neck) were compensated using beam attenuators or mid-line shielding, to restrict the oesophageal dose to less than 42 Gy. CT planning was not used routinely, unless there was concern regarding dose heterogeneity related to changes in contour, for example, proceeding from chest to neck, where the dose to the oesophagus could potentially be excessive or where the target volume required CT for accurate delineation. (4) Check films in weeks 1 and 2 of radiotherapy. If week 2 film was satisfactory, no further check films are required, otherwise repeat weekly.
2. Concurrent chemotherapy: During the external radiation therapy all patients received chemotherapy comprising of Cisplatin intravenous (i.v.) weekly and Vinorelbine i.v. days 1, 8, 22. Day 15 Vinorelbine was excluded due to the expected cumulative neutropenia from the prior doses. The doses of each agent were escalated through six planned dose levels (DLs). Vinorelbine was escalated from 15 to 30 mg m^−2^ week^−1^ and Cisplatin from 20 to 30 mg m^−2^ week^−1^. All chemotherapy was given within 2 h of the delivery of the radiation fraction on that day. Premedication was standard. No pre- or post-hydration was utilised.
3. Definition of dose-limiting toxicities (DLTs): These were based on toxicities experienced during, and within, 2 weeks following CRT, and are defined as follows: (1) grade 4 neutropenia (ANC<0.5 × 10^9^ l^−1^) of any duration; (2) grade 4 thrombocytopenia (platelet count<10 × 10^9^ l^−1^) or grade 3 thrombocytopenia (platelet count 10–49 × 10^9^ l^−1^) with bleeding; (3) febrile neutropenia; (4) grade 3 or 4 nonhaematological toxicity (within or outside the radiation field), including nausea and vomiting despite adequate antinauseant therapy; (5) interruption of radiotherapy in excess of 1 week; (6) chemotherapy omitted for 1 or more weeks; (7) toxicity requiring one or more dose reductions during CRT.
4. Dose escalation schema: Three patients were planned to be entered into each DL. If no DLTs were observed in these three patients, the next DL was opened. If DLTs were observed in ⩾2 of three patients, then no further dose escalation took place. If DLTs were observed in one of three patients, then three additional patients (total of six patients) were accrued at this level. If DLTs were observed in ⩽1 of six patients, then the next DL was opened. If DLTs were observed in ⩾2 of six patients, no further dose escalation took place. The highest administered dose was defined as that DL in which two or more of three or six patients had DLTs. The recommended DL (one below the highest administered dose) reached was expanded to a total of 15 patients to obtain further data concerning acute and late toxicities and response.
5. Dose modifications during CRT: Radiotherapy, together with chemotherapy, was suspended if the patient experienced grade 3 or 4 radiation-associated toxicities (e.g. oesophagus, lung, skin, heart). RT only was recommenced once reactions had improved to grade 1 or better within a maximum of 2 weeks. During CRT, the chemotherapy doses were modified based on the worse grade of toxicities. Treatment modification was in two forms: either (a) permanent dose reductions or (b) treatment deferral with the recommencement at a reduced dose on recovery, that is, neutrophils ⩾1.0 × 10^9^ l^−1^ or platelets >50 × 10^9^ l^−1^, and nonhaematological toxicity to grade 1. Patients entered at DL 1 requiring more than one dose reduction or entered at higher DLs requiring more than two dose reductions discontinued chemotherapy; however, the radiotherapy continued. Treatment was stopped early due to either disease progression, a greater than 2-week delay in radiotherapy delivery, unacceptable toxicity, patient request or patient noncompliance with the required investigations.

#### Consolidation chemotherapy

At 4 weeks after completing CRT, all patients were planned to receive two cycles of chemotherapy, unless there was radiological progressive disease on restaging prior to this time. The chemotherapy consisted of Cisplatin 80 mg m^−2^ i.v. day 1 and Vinorelbine 25 mg m^−2^ i.v. weekly by three, the cycles were repeated every 28 days. The toxicities from these cycles were not used to determine the DLTs for CRT. Premedication and hydration regimens for Cisplatin and chemotherapy dose modifications were standard. On day 1 of each cycle, the following parameters were required: ANC⩾1.5 × 10^9^ l^−1^, platelet count⩾100 × 10^9^ l^−1^ and creatinine clearance⩾55 ml min^−1^, and all nonhaematological toxicities resolved to at least grade 1. If these were not achieved within 2 weeks, the patient ceased treatment.

### Monitoring procedures and tests

At baseline (within 2 weeks of trial entry), patients had performance status recorded, bloods taken for full blood examination and differential and biochemistry (including serum urea, electrolytes and creatinine, calcium, liver function tests (bilirubin, AST/ALT, ALP, GGT)) and creatinine clearance estimation (using radioisotope renal scan or derived from serum creatinine using Cockcroft–Gault formula). Staging comprised of a chest X-ray PA/lateral, CT scan of the chest and upper abdomen, CT scan of the brain and bone scan if clinically indicated, FDG-PET scan (optional, subject to availability), spirometry and the assessment of DRS by the Lung Cancer Symptom Scale (LCSS) (Hollen *et al*, 1993).

During treatment patients were reviewed weekly, with documentation of acute toxicities. Bloods were taken weekly as above, except for haematology, which was performed twice weekly during CRT. Creatinine clearance was estimated weekly during CRT and on day 1 of each course of consolidation chemotherapy. DRS were assessed weekly during CRT.

Tumour response (radiological and FDG-PET), spirometry and completion of the LCSS were repeated at 3 weeks post-CRT and 4 weeks post-completion of all therapy. Patients were then reviewed every 2 months for late radiation toxicities (RTOG/EORTC criteria), and restaging, (until documented progression or earlier if clinical suspicion) until death or loss to follow-up.

### Statistical analysis

#### Criteria for the assessment of treatment outcomes and toxicities

Tumour response: Radiological response was assessed as above, utilising the same methods as at baseline. The best overall radiological response within and outside the radiation field was documented using the WHO criteria (Miller *et al*, 1981).
Toxicities: Acute toxicities were assessed using the NCI Common Toxicity Criteria (version 2.0, 30 April 1999). Late radiation toxicities were assessed using the RTOG/EORTC criteria.

#### Statistical methods

The worst grades of acute toxicities experienced during CRT, which were considered to be definitely or probably related to protocol treatment, were reported for all patients by DL using descriptive statistics. The overall response rate (complete (CR)+partial responses (PR)) following CRT was estimated as the percentage of all patients, and its 95% confidence interval was estimated using the exact probabilities of the binomial distribution.

Disease-related. symptoms were assessed by the LCSS (Hollen *et al*, 1993). The patient subjective rating scale was used in these analyses and the data analysed as per the published methodology (Hollen *et al*, 1993). A Wilcoxon signed-rank test was used to find the difference in symptom scores before and after radiotherapy treatment. A two-sided *P*<0.05 was considered to be statistically significant.

All patients who commenced treatment were included in the analyses of PFS and OS. Patients were followed to a close-out date of 29 September 2003. Progression-free survival time was measured from the date of commencing protocol treatment to the date of first progression (local, regional of distant) or death without previous progression. Overall survival time was measured from the date of commencing protocol treatment to the date of death from any cause. The Kaplan–Meier method was used to estimate OS and PFS, with censoring of survival times at the close-out date. The Brookmeyer–Crowley method was used to estimate 95% confidence intervals for median survival times. The 95% confidence intervals for the percentages surviving at particular times were calculated using the logit transformation.

A potential follow-up time for each patient was defined as the time from commencing protocol treatment to the close-out date, unless the patient was lost to follow-up. A competing risks analysis was used to estimate cumulative incidence rates for different types of first progression.

## RESULTS

### Patients

In all, 24 patients were recruited from 30 June 2000 and 16 September 2003, from two oncology centres, across four DLs. Patient demographics are summarised in Table 1. Males predominated, with over 63% of patients being over 60 years of age. The majority, 22 patients (92%), had stage IIIB or limited stage IV disease. Approximately 70% of patients had disease incorporated within the field due to mainly N3 disease (supraclavicular or contralateral hilar or mediastinal nodal involvement), while two patients had less extensive disease, but with adverse clinical or prognostic factors contraindicating radical therapy.

### Treatment delivery

#### Chemoradiotherapy

1. Chemotherapy: A total of 24 patients were accrued across four DLs of weekly Cisplatin plus Vinorelbine: DL 1 (15, 20 mg m^−2^, respectively), three patients; DL 2 (20, 20 mg m^−2^), three patients; DL 3 (25, 20 mg m^−2^), 15 patients; DL 4 (30, 20 mg m^−2^), three patients. Dose level 4 was identified as the highest administered dose and hence DL 3, as discussed above, was expanded to a total sample of 15 patients. Full dose delivery was achieved in DL 1 and 2, but dose omissions were observed in DL 3 and 4, specifically on days 15 and 22. The planned day 15 Cisplatin treatment was omitted in six patients due to the following reasons: grade 4 neutropenia in five (two in DL 4 and three in DL 3) and chest infection in one patient (DL 3). Vinorelbine was not planned to be administered on day 15. On day 22, both drugs were omitted in two patients in DL 3, due to grade 3 neutropenia and persistent chest infection, respectively. At the recommended DL, DL 3, the relative dose intensity (ratio of actual dose to planned dose) for the weekly Vinorelbine was 92% (range 66.7–100%) and for Cisplatin was 88.3%, (range 50–100%).
2. Radiotherapy: The planned total dose of 40 Gy was delivered to 23 or 96% of patients, with the remaining patient, receiving 30 Gy in 15 fractions, having refused to continue with treatment. In all, 19 or 79% of the patients completed the prescribed radiotherapy over the planned 28 days (5 days per week, weeks 1–4). The reasons for a break in the planned treatment of five patients were: two patients with a febrile episode (nonfebrile neutropenia), one patient unwell, one patient unknown and one patient with refusal to continue.
3. Dose-limiting toxicities: These were prospectively defined and assessed only during the CRT dose escalation component of the treatment, as described above. The DLTs observed were haematological, where, in DL 4, two of the three patient cohort developed grade 4 neutropenia. Hence, DL 4 was determined to be the highest administered dose and DL 3 was defined as the recommended dose for expansion to 15 patients in total.

#### Consolidation chemotherapy

Of the 24 patients entered, 20 had cycle 1 and 16 patients had both cycles 1 and 2. Four patients had not received the planned first courses of consolidation therapy due to the following reasons: progressive disease (one patient), persistent chest infection (one patient), withdrawal of consent (one patient) and inadequate haematological recovery (one patient). Of the 20 patients who planned to receive the second course, four had not received therapy due to: persistent nausea and vomiting (one patient), febrile neutropenia (one patient), acute renal impairment (one patient) and unknown (one patient). The dose intensity overall for Cisplatin on day 1 was 96.2% and the Vinorelbine (days 1, 8, 15) was 73%.

### Toxicity

The haematological and nonhaematological toxicities from day 1 of CRT to the commencement of the first course of consolidation chemotherapy are detailed in Tables 2 and 3, respectively. The concurrent therapy was well tolerated overall. There were four patients who developed grade 4 neutropenia; however, only one developed febrile neutropenia. A further patient developed a chest infection. In terms of the nonhaematological toxicities, of note, there were no reported cases of grade 3 or 4 radiation oesophagitis or pulmonary toxicity.

In the 20 patients who proceeded onto consolidation chemotherapy, grade 4 neutropenia was observed in seven (35%) patients, two patients each in DLs 2 and 4 and three in DL 3. Febrile neutropenia was observed in two patients (10%), one each in DL 1 and 3, respectively. Other toxicities included sensory neuropathy, grade 2 in one patient in DL3, and grade 1 in two patients, one each in DLs 1 and 4. Grade 3 renal impairment was observed in one patient in DL3.

The following late radiation toxicities, according to the EORTC/RTOG criteria, were observed: (i) oesophageal: grade 1, one patient, DL1; grade 2, one patient, DL3; (ii) skin: grade 1, one patient, DL2 and (iii) lung: grade 1, one patient, DL1; grade 3, two patients, DL 1 and 3, respectively.

Spirometry was performed in patients at baseline (*n*=24), at 3 weeks post-CRT (*n*=18) and at the completion of all therapy (*n*=11). The mean FEV_1.0_ at each of these time points was: 70.9% predicted (range 38–101%), 74.5 and 62%, respectively. The FVC at each of these time points was 86.2% predicted (range 51–124%), 91.8 and 82%, respectively.

### Response

The overall radiological response data following the completion of all therapy (both within and outside the radiation field) are summarised in Table 4. Of the 24 patients entered, 23 were evaluable for response, with one patient refusing to continue treatment during CRT. Thus, of these 23 patients, the overall radiological response rate was 65% (15 of 23) (95% confidence interval (CI): 34–77%), with CR and PR rates of 4% (one of 23) and 61% (14 of 23), respectively. Stable disease was observed in 17% (four of 23), with progressive disease in two patients (9%), one outside and one inside the radiation field.

The radiological response rate within the radiation filed is also summarised in Table 4, with similar results as above. In all, 30% or eight patients had sites of disease that were not incorporated within the radiation field. Of these, a PR was observed in one patient and two patients had progressed outside the radiation field.

### Sites of relapse

**Figure 1:**
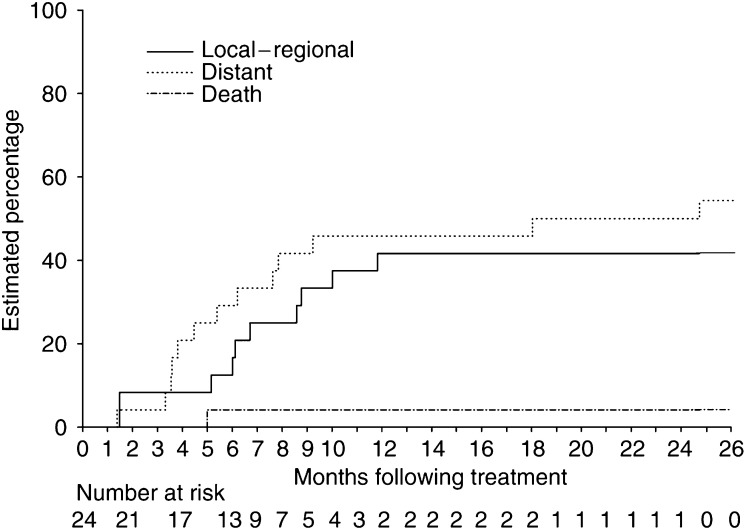
The cumulative incidence of first progress.

The location of first progression is detailed in Table 5. Of the 24 patients entered, local control was observed in 18 patients; however, regional nodal relapse (within or adjacent to the radiation field) was observed in 12 patients, with half being isolated without progression at other sites. Distant progression was observed in 13 (54%) patients; in the majority of cases this was isolated without local or regional involvement. One patient had died without progression. The cumulative incidence of first progression is shown in Figure 1. The cumulative incidence of local–regional first progression at 6 and 12 months was 12.5 and 41.7%, respectively, and for distant progression (alone or with locoregional progression) the risk was 29.2 and 45.8%, respectively.

### Survival parameters

**Figure 2:**
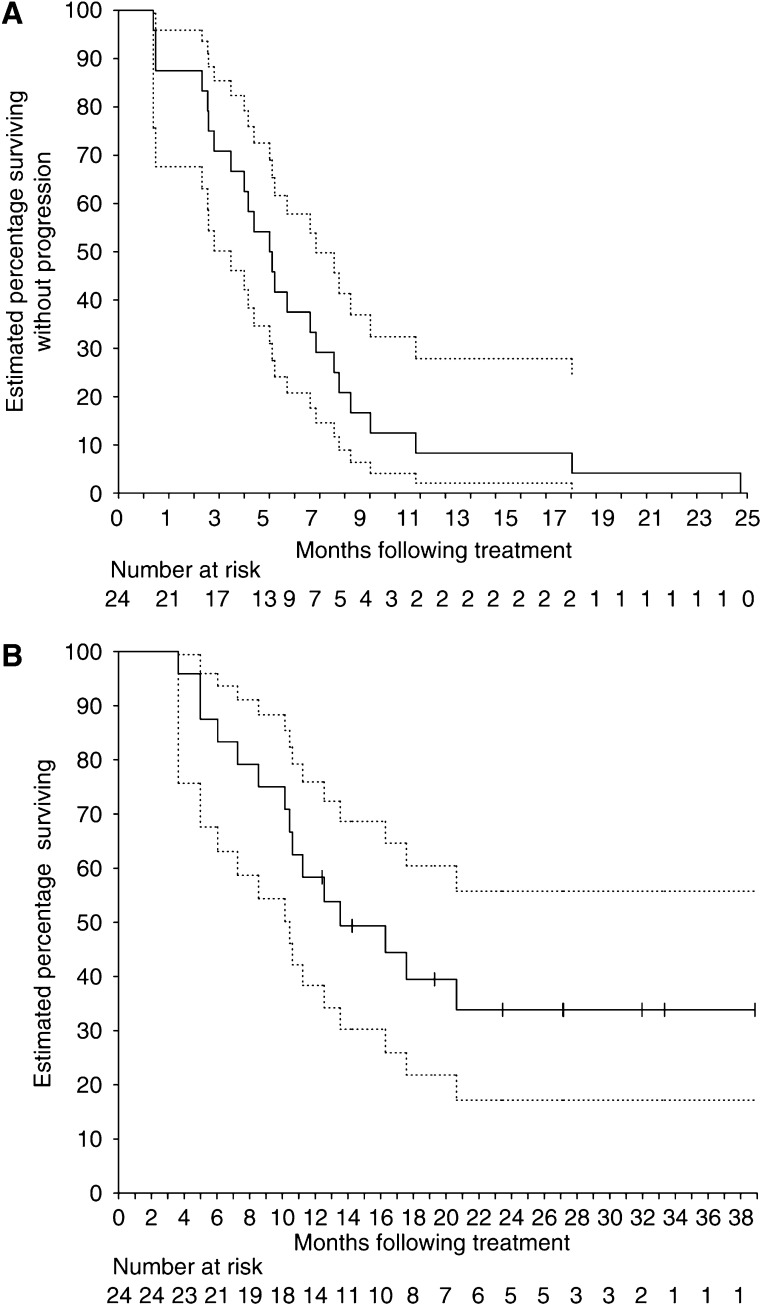
(**A**) Progression free-survival for all patients. (The hatched line represents the 95% CI.) (**B**) The OS curve for all 24 patients. (The hatched line represents the 95% CI.)

Patients were followed up from commencing protocol treatment to a close-out date of 29 September 2003. One patient was lost to follow-up. The median potential follow-up time was 21.6 months (range 12.4–38.9 months), with all patients having progressed or died at the close-out date. The Kaplan–Meier PFS and OS survival curves are shown in Figure 2A and B. The actuarial PFS was 6.1 months (95% CI 4.5–7.9 months), with the estimated PFS at 6 and 12 months being 54% (95% CI 35–73%) and 8% (95% CI 2–28%), respectively.

The actuarial OS was 13.5 months (95% CI 10.4–>38.9 months). The OS at 6, 12 and 24 months was 88% (95% CI 68–96%), 58% (95% CI 38–76%) and 34% (95% CI 17–56%), respectively.

Of the 24 patients, 12 were classified as stage IIIB and 10 stage IV. The median OS for the stage IIIB and IV patients was 16.3 (95% CI 10.4–>38.9 months) and 12.4 months (95% CI 5.0–>27.1 months), respectively. The OS difference between the stages was not significant (*P*=0.358).

### DRS and QOL

Of the 24 patients entered, only 20 patients had LCSS data at baseline and 19 patients at 3 weeks post-CRT. The major presenting symptoms are summarised in Table 1, with those due to local disease, including cough, pain and dyspnoea, being recorded by at least 70% of patients. Over 80% of patients had four or more symptoms at baseline, with 30% having six symptoms.

**Figure 3:**
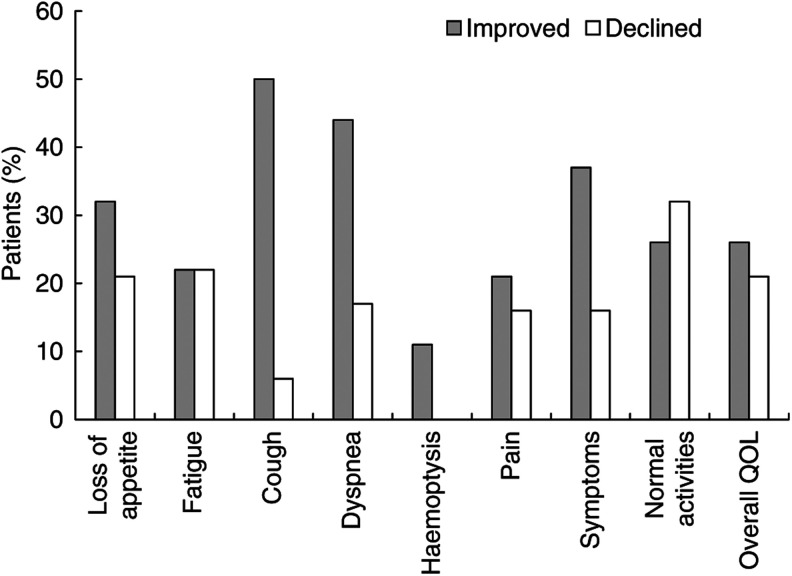
Change in LCSS scores between baseline and 3 weeks after CRT. (Improved: >10 mm better score after CRT. Declined: >10 mm worse score 3 weeks after CRT.)

As shown in Figure 3, the post-CRT LCSS evaluation found that 30–50% of patients had an improvement in loss of appetite, cough and dyspnoea, with 37% of patients showing an improvement in overall symptom distress. A decline in scores was observed in up to 32% of patients subject to the parameters; for example, no patient had a decline or worsening of haemoptysis, but for 32% there was a deteriorating normal activity. A stabilisation of LCSS scores post-CRT was observed in 39–90% of patients, subject to the parameter.

Relative to baseline, at 3 weeks post-CRT, there was a nonsignificant trend towards improvement in scores for loss of appetite (*P*=0.20), dyspnea (*P*=0.13), haemoptysis (*P*=0.14), pain (*P*=0.36), overall symptomatic distress (*P*=0.19) and overall QOL (*P*=0.54) after CRT (Table 6). There was a statistically significant improvement in cough (*P*=0.02) following CRT. The average LCSS score decreased after CRT (27.1 *vs* 21.2, *P*=0.08).

## DISCUSSION

Patients with unresectable NSCLC and good PS who are unable to receive radical combined CRT may be treated initially with chemotherapy alone or with HDPR if there are significant local symptoms. The incremental benefit from the addition of chemotherapy to HDPR has not been defined. It however represents an important ground for investigation (Bogart, 2004), given the benefits achieved by its incorporation within the radical radiation setting.

The primary aim of this trial was therefore to identify a tolerable regimen of weekly Cisplatin and Vinorelbine combined with HDPR (40 Gy) that could be subsequently compared to radiation alone in a randomised trial. The eligibility criteria in this study represented a heterogeneous group of patients, but all patients were at least ECOG 1 and most were not suitable for radical radiotherapy due to the extent of disease (i.e. 50% with stage IIIB and 42% stage IV disease and 30% having disease outside the irradiated volume). All patients were locally symptomatic, requiring palliation, with the majority having multiple DRS at presentation.

Of the entire cohort, 20 patients were staged by FDG-PET. This imaging mode has been shown by several studies to upstage approximately 30% of patients being considered for radical therapy by the detection of unsuspected metastasis. It has also been shown to provide a better correlation with survival *vs* conventional staging (Dunagan *et al*, 2001; Kalff *et al*, 2001; Hoekstra *et al*, 2003; MacManus *et al*, 2003). A retrospective study from our institution has also demonstrated that OS correlated with FDG-PET defined metastatic disease burden, for example, 12 months in patients with one metastatic site *vs* 5 months if greater than one site (Hicks *et al*, 2001; MacManus *et al*, 2003). On the other hand, the use of PET staging here may have led to selection bias, identifying those patients with reduced disease burden outside the chest and hence better OS relative to those staged by standard imaging as used in older studies.

The selection of patients suitable for this novel combined approach was rigorous and consistent. The inability to deliver radical CRT either for technical (extent of thoracic disease or presence of distant disease) or clinical reasons (medical comorbidities) was based on consensus following detailed discussions of the patient's case among the same cohort of medical specialists. These included thoracic radiation and medical oncologists, as well as the input of radiologists, nuclear and respiratory medicine physicians within the same tertiary referral cancer centre.

The regimen as detailed was both tolerable and showed promising activity. There was dose escalation of Vinorelbine and Cisplatin during HDPR, and the toxicities observed here defined DL 3 for evaluation in subsequent trials. The prespecified DLTs were pragmatic and clinically relevant to the treatment of such patients with a combined approach. The DLTs identified were as expected haematological, that is, grade 4 neutropenia, but overall there was only one episode of febrile neutropenia and no grade 3 radiation oesophagitis. Of note, there were no significant late oesophageal toxicities or treatment-related deaths.

The overall dose intensity of chemotherapy during CRT was approximately 90% and also in the recommended DL (DL 3), with all but one patient having received the full radiation dose. The consolidation therapy of two cycles of Cisplatin and Vinorelbine was delivered to 16 of the 24 patients overall; the toxicities observed were not unexpected for this regimen, being mainly haematological in nature. The current role of consolidation chemotherapy following either high-dose palliative or radical CRT has not been established. Two phase II trials, one of which was a three-arm randomised study, have suggested an improved survival with the addition of consolidation chemotherapy to radical CRT relative to CRT alone (Choy *et al*, 2002; Gandara *et al*, 2003). Within the limits imposed by a phase I trial design, the regimen has demonstrated promising efficacy, with a overall radiological response rate (in and out of field) of 65% (15/23) comprising of CR in 4% (one) and PR in 61% (14).

In the literature, there have been few studies that have addressed the issue of chemotherapy in combination with palliative radiotherapy in patients with NSCLC. In contrast to this trial, these studies have utilised hypofractionated radiation schedules with differing chemotherapy regimens. The first assessed the combination of weekly Docetaxel (10–45 mg m^−2^ week^−1^) with 50 Gy, 5 Gy per week in 26 patients with stage III/IV disease. The response rate in 19 evaluable patients was 73.7%, with the remainder having stable disease. No local recurrence was observed with this regimen. The toxicities were mainly chemotherapy-related, but palliative benefit in terms of symptom response was not recorded (Schwarzenberger *et al*, 2004).

A further study evaluated 50 patients with stage IV disease treated with Carboplatin (300 mg m^−2^, days 1 and 29) plus oral Etoposide (50 mg m^−2^, days 1–29) and concurrent radiotherapy 28 Gy, 14 Gy fractions at one per week (days 1 and 8). The overall response rate was 28% with a median survival of 7 months. Nearly one-fifth of the patients had grade 3 oesophagitis and 9% had grade 3 pulmonary toxicity. Approximately 60–75% had an improvement in their local symptoms; however, a validated symptom or QOL scale was not used (Jeremic *et al*, 1999).

A more recent phase I study in 29 patients evaluated two such regimens as follows: (i) hypofractionated radiotherapy, 17 Gy, 8.5-Gy fractions at one per week, combined with Vinorelbine, from 20 to 30 mg m^−2^ on days 1 and 8 every 28 days, and (ii) 60 Gy, 5-Gy fractions at one per week, plus weekly Vinorelbine from 10 to 20 mg m^−2^, for 12 weeks. In the first regimen the DLTs were haematological, with one treatment-related toxic death, and in the second one patient suffered grade 3 oesophagitis and one had a late toxic death due to pneumonitis. Response data were not provided (Slivano *et al*, 2000).

It hence appears that the regimen reported here is possibly as efficacious as the hypofractionated CRT schedules, albeit with the absence of significant toxicities, most likely reflecting the reduced fraction size. The more important question remaining is whether the addition of chemotherapy to HDPR provides additional benefit to HDPR alone or to chemotherapy alone in these patients.

It is usual for patients with stage IIIB and IV patients (making up 92% of our cohort) to be treated with chemotherapy alone rather than external beam therapy. In the majority of phase III studies, stage IIIB contribute 10–40% (Frasci *et al*, 2000; Ranson *et al*, 2000; Kelly *et al*, 2001; Scagliotti *et al*, 2002; Schiller *et al*, 2002) of patients and stage IV patients would vary considerably in disease bulk outside the thorax (Frasci *et al*, 2000; Ranson *et al*, 2000; Scagliotti *et al*, 2002). Systemic therapy is associated with a median survival ranging from 6 to 8 months. As discussed above, the trial methodology and staging methods used in this study would preclude a direct comparison to chemotherapy-alone trials. However, local control is still relevant in patients treated with chemotherapy alone, with up to 10–50% requiring local palliative radiation. (Anderson *et al*, 2000; Ranson *et al*, 2000).

While considering the above limitations, it would be of interest to compare the results reported here with those from randomised trials of palliative radiation summarised in Table 7. These studies are heterogenous in terms of the eligibility criteria, assessment of symptomatic benefit, treatment and staging procedures, the latter reflecting that some of these were reported in the 1980s. In comparison to our own study, similar patient cohorts and radiation schedules were assessed in the trials reported by the RTOG (Simpson *et al*, 1985) and the MRC (Macbeth *et al*, 1996). The overall radiological response and OS observed in the study reported here are comparable, and in particular may approach schedules with higher biological effective doses. However, it must be stated that the OS may have been impacted upon by selection bias generated by FDG-PET staging and also the influence of subsequent modern chemotherapy on progression.

Similar observations may be inferred by comparing the relapse patterns from this study and those of the reported trials. In this study local progression was observed in 25% of patients, with regional nodal relapse being observed in 42%, the majority being isolated, perhaps indicating geographical misses or areas not attaining full radiation dose. Subject to the variation in the definition of relapse sites and imaging across studies, these results appear promising relative to the reported randomised trials of HDPR. In the RTOG study (Simpson *et al*, 1985) for the entire cohort, distant and local relapses were reported in 52 and 59%, respectively. Local relapse ranged from 49% for the 40-Gy split course group to 67% for the 40-Gy continuous cohort (Simpson *et al*, 1985). In the MRC study, the first site of definite/suspected relapse was the primary site in 33% of the F2 and 38% of the F13 patients, and for distant sites 39% in both (Macbeth *et al*, 1996).

Patient LCSS was measured prior to commencement of treatment, during and 3 weeks post-completion of CRT. Post-CRT, there was a significant improvement in cough (*P*=0.02), as well as a nonsignificant trend towards improvement in the other symptoms and parameters assessed. There was no decrement in overall QOL despite the rather intense treatment. The palliative benefits reported in our trial are consistent with the reported literature subject to dose and fractionation. The level of benefit relative to the these studies may have been underestimated due to the short time interval from treatment completion to the final assessment of symptom benefit (Simpson *et al*, 1985; Macbeth *et al*, 1996; Sundstrom *et al*, 2004). In the MRC study (Macbeth *et al*, 1996), symptom response was assessed by the Rotterdam Symptom Checklist for the duration of the trial. The percent of patients with palliation for cough by 1 *vs* 2 months post-treatment was 36 *vs* 48%, respectively, with the other symptoms having improved by a similar trend over this time. The benefit had actually increased by an additional 14% of cases at 3 months (Macbeth *et al*, 1996).

In conclusion, we have therefore defined a regimen for weekly Cisplatin plus Vinorelbine combined with HDPR in patients with NSCLC not suitable for radical CRT, but of good performance status and requiring local palliation. The regimen is both tolerable with an interesting level of efficacy in terms of radiological and functional response, as well as palliative benefit. The regimen will be evaluated further as part of a national study.

## Figures and Tables

**Table 1:** Patient characteristics

**Characteristics**	**Number**	**%**
*Sex*		
Male : female	14 : 10	58 : 42
		
*Age*		
Median	65	
Range	41–75	
		
*Histology*		
Squamous	8	33
Adenocarcinoma	8	33
Other	8	33
		
*Stage*		
I (T2N0)	1	4
IIIA (T3N2)	1	4
IIIB (T1–3,N3 or T4,N0–3)	12	50
IV (T1–4,N0–3,M1)	10	42
		
*Distribution of M1 sites (n=10)* a		
Bone	4	17
Lung (different lobe to primary)	3	12
Adrenal	2	8
Distant lymph node	2	8
Pericardium	1	4
Pleura	1	4
		
*Disease anatomy in relation to radiation field*		
Disease entirely inside field	16	70
Disease inside/outside radiation field	8	30
		
*Weight loss over prior 3 months*		
None	15	63
⩽10%	9	38
		
*ECOG performance status*		
0	4	17
1	18	75
2	2	8
		
*Presenting lung cancer symptoms as recorded by the LCSS (n=20)* b		
Patients with ⩾4 symptoms	17	85
Dyspnoea	17	85
Haemoptysis	7	35
Cough	17	85
Pain	14	70
Fatigue	18	90
Anorexia	17	85

a Patients may have had more than one site of M1 disease.

b Those who had symptoms ⩾5 mm in the symptom scale were considered as symptom occurrence.

**Table 2:** Haematological toxicities observed during concurrent chemoradiotherapy (NCI-CTC Version 2, 30 April 1999)

		**Dose levels**				
**Toxicities**	**Worst grade**	**1 (*n*=3)**	**2 (*n*=3)**	**3 (*n*=15)**	**4 (*n*=3)**	**Total (%)**
Leucocytes	3	0	0	4	3	7 (29)
	4	0	0	2	0	2 (8)
						
Neutrophils	3	0	0	2	0	2 (8)
	4	0	0	2	2	4 (17)
						
Platelets	3	0	0	0	0	0 (0)
	4	0	0	0	0	0 (0)
						
Haemoglobin	3	0	0	0	0	0 (0)
	4	0	0	0	0	0 (0)

**Table 3:** Nonhaematological toxicities observed during concurrent chemoradiotherapy (NCI-CTC Version 2, 30 April 1999)

		**Dose levels**				
**Toxicities**	**Worst grade**	**1 (*n*=3)**	**2 (*n*=3)**	**3 (*n*=15)**	**4 (*n*=3)**	**Total (%)**
Febrile neutropenia	3	0	0	1	0	1 (4)
Fever without grade 3 or 4 neutropenia	3	0	0	1	0	1 (4)
Vomiting	2	0	0	2	0	2 (8)
Fatigue	3	0	1	2	0	3 (13)
Weight loss	2	0	1	0	0	1 (4)
Inner ear/hearing	2	1	0	0	0	1 (4)
Renal impairment	1	0	0	1	0	1 (4)
Radiation dermatitis	2	1	0	4	1	6 (25)
Radiation oesophagitis	2	1	1	5	1	8 (33)

**Table 4:** Radiological response following the completion of all therapies

	**Dose levels**				
**Response parameters** **(*n*=23)**	**1 (*n*=3)**	**2 (*n*=3)**	**3 (*n*=14)**	**4 (*n*=3)**	**No. (%)**
*Overall best response*					
Complete response	0	0	1	0	1 (4%)
Partial response	2	3	7	2	14 (61%)
Stable disease	0	0	4	0	4 (17%)
Progressive disease	1	0	0	1	2 (9%)
Not evaluable	0	0	2	0	2 (9%)
					
*Best response in radiation field*					
Complete response	0	0	1	0	1 (4%)
Partial response	2	3	7	2	14 (61%)
Stable disease	0	0	4	0	4 (17%)
Progressive disease	0	0	0	1	1 (4%)
Not evaluable	1	0	2	0	3 (13%)

**Table 5:** The location of first progression

**Location**	***N***	**% (*n*=24)**
Local^a^	2	8.3
Regional^b^	6	25.0
Distant^c^	9	37.5
Local–regional	2	8.3
Regional–distant	2	8.3
Local–regional–distant	2	8.3
Death without progression	1	4.2

a Primary lesion.

b Relapse in (a) nodal stations within the radiation field, that is, involved hilar, mediastinal or supraclavicular nodes or (b) mediastinal or supraclavicular nodes adjacent to the radiation field.

c M1 sites.

**Table 6:** The descriptive statistics of the LCSS scores at baseline and at 3 weeks post CRT

	**Baseline (*n*=20)**		**3 weeks after CRT (*n*=19)**	
**Item**	**Mean (s.d.)**	**Median (range)**	**Mean (s.d.)**	**Median (range)**
Loss of appetite	27.7 (24.5)	24 (0, 99)	23.1 (27.2)	9 (0, 91)
Fatigue^a^	35.5 (27.8)	31 (0, 95)	33.8 (27.2)	27 (0, 85)
Cough^a^	33.4 (27.9)	25 (0, 87)	16.7 (19.7)	12 (0, 83)
Dyspnea^a^	38.9 (31.3)	33 (0, 93)	23.1 (25.0)	15 (0, 80)
Haemoptysis	4.3 (5.1)	2 (0, 18)	2.5 (2.9)	1 (0, 8)
Pain	19.0 (22.3)	11 (0, 89)	14.4 (18.1)	11 (0, 65)
Overall symptomatic distress	31.5 (31.8)	19 (0, 93)	22.4 (22.6)	13 (0, 79)
Normal activity	26.7 (23.9)	18 (0, 97)	29.7 (30.9)	12 (0, 98)
Overall quality of life	26.7 (14.8)	18 (0, 85)	25.4 (25.8)	11 (0, 90)
Mean LCSS	27.1 (16.6)	28 (2, 62)	21.2 (15.6)	19 (1, 50)

a *N*=18 at followup assessment.

**Table 7:** Summary of the randomised trials of high-dose palliative radiotherapy in patients with NSCLC

**Reference**	**Patient cohort**	**Treatment arms**	**ORR**	**Local symptom palliation**	**Median OS (mos)**
Michael *et al*	24 Stage 1 – limited IV, local symptoms	Cis+Vin/40 Gy/20#s/5 pw → Cis+Vin × 2	65%	21–50%	13.5
					
Nestle *et al* (2000)	121 Stage III^a^, and 31 limited stage IV^a^	60 Gy/30#s/5 pw 32/16#s/10 pw	66% NS	Upto 33% NS	8.4
					
Reinfuss *et al* (1999)	240 Stage III^a^	50 Gy/25#/5 pw 40 Gy/10#/5^b^ pw (60)^c^ Delayed:20 Gy/5#/5 pw (30)			12 9 6
					
Macbeth *et al* (1996)	509^a^ No distant disease	17 Gy/2#s/1 pw (44.6) 39 Gy/13#s/5 pw (48.7)	ND	45–89% at 3 months	7 9
					
Sundstrom *et al* (2004)	421, Stage III, IV,^a^ local Sx	17 Gy/2#s/1 pw (44.6) 42 Gy/15#s/5 pw (50.4) 50 Gy/25#s/5 pw	ND	20–90% NS	8.2 7 6.8
					
Teo *et al* (1988)	273^d^, Stage III or IV	45 Gy/18#s/5 pw (50.6) 31.2 Gy/4#s/1 pw (76.4)	53%^e^ 50%	71%^f^ 54% (*P*<0.02)	4.6 NS
					
Simpson *et al* (1985)	409, T4NxM0, TxN3M0	40 Gy/10#s/5 pw, split course (60) 30 Gy/10#s/5 pw (37.5) 40 Gy/20#s/5 pw Post RT → Chemo *vs* Observation	40%^g^ 44% 42% NS	Overall, 24.5% Sx free & 46.9% ↓ Sx intensity	6.2 6.4 6.9
					
Gaze (2001)	148, Stage ND	30 Gy/10#s/5pw (37.5) 10 Gy/1# (30)	ND	70.1% 46.7%	6.4 5.3 NS
					
Abratt *et al* (1995)	84, Stage 3 (primary disease >6 cm or >1 mediastinal node	35 Gy/10#s/5 pw (48.1) 45 Gy/15#s/5 pw (56.2)	56%^e^ 51%	68%^h^ 76%	8.5 8.5

Chemo=chemotherapy; Cis=cisplatin; NE=not evaluated; NS=no significant difference between arms; ND=not documented; Obs=observation; pw=per week; RT=radiotherapy; Sx=symptoms; Vin=vinorelbine.

a Patients unsuitable for radical approaches.

b Two courses of 20 Gy/5#s per week separated by 4 weeks.

c Biological effective dose, based on an *α* : *β* of 2 Gy.

d Patients with bilateral pulmonary disease, N3 disease, malignant effusion, bulky tumour or nodes >8 cm rendering radical therapy undeliverable, chest wall infiltration with rib erosion, extrathoracic metastases.

e Chest X-ray assessment of response.

f Symptom response if disappearance of all Sx, or reduced severity or frequency of one or more Sx without the emergence of new intrathoracic symptoms.

g Radiotherapy alone arms only.

h Complete and partial symptom response rate.
